# Use and Acceptance of Innovative Digital Health Solutions Among Patients and Professionals: Survey Study

**DOI:** 10.2196/60779

**Published:** 2025-05-08

**Authors:** Fritz Seidl, Florian Hinterwimmer, Ferdinand Vogt, Günther M Edenharter, Karl F Braun, Rüdiger von Eisenhart-Rothe, AG Digitalisierung der DGOU, Peter Biberthaler, Dominik Pförringer

**Affiliations:** 1 Department of Orthopaedics and Sports Orthopaedics, Klinikum rechts der Isar, Technical University of Munich, TUM University Hospital, Ismaninger Str. 22, Munich, 81675, Germany, 49 89-4140-1063; 2 Institute for AI and Informatics in Medicine, Technical University of Munich, Munich, Germany; 3 Department of Cardiac Surgery, Artemed Clinic Munich South, Munich, Germany; 4 Department of Cardiac Surgery, Paracelsus Medical University, Nuremberg, Germany; 5 Department of Anaesthesiology, Klinikum rechts der Isar, Technical University of Munich, TUM University Hospital, Munich, Germany; 6 Department of Trauma Surgery, Klinikum rechts der Isar, Technical University of Munich, TUM University Hospital, Munich, Germany

**Keywords:** eHealth, digital health, medical data use, information, survey, adoption

## Abstract

**Background:**

Digital solutions are gaining increasing importance and present a challenge regarding their introduction and acceptance into professional medical environments. Significant advances have been made regarding the availability, safety, and ease of use of data generated by a multitude of devices and wearables. However, data security and data protection are delaying factors. The underlying analysis focuses on the use and acceptance of digital solutions, and their respective differences between health care professionals and patients.

**Objective:**

This study examines the current use and acceptance of digital solutions among health care professionals and patients. In addition, it derives an outlook on future developments and expectations in the setting of innovative technologies able to penetrate the health market.

**Methods:**

An anonymous web-based survey of 23 multiple-choice and 3 open-text questions was conducted among medical professionals and patients between April and September 2023. In this study, quantitative analysis was performed using Python, with Pandas for data processing and Matplotlib for visualization. Chi-square tests were used to analyze binary categorical data, while Mann-Whitney *U* tests were used to evaluate ordinal data. Additionally, a qualitative analysis was conducted to summarize the results of the open-ended questions.

**Results:**

During 178 days, the survey garnered 2058 clicks, resulting in 1389 participants (67.5% response rate). A total of 1002 participants completed the entire questionnaire, while 387 (27.9%) did not finish. Incomplete responses were excluded from the comprehensive analysis. The sample comprised 271 (27%) physicians and 731 (73%) patients. The study found significant agreement between both groups in adopting and foreseeing the use of digital health tools and telemedicine. Both groups recognized the future importance of digital health without substantial differences.

**Conclusions:**

Overall, attitudes toward digital health and telemedicine were consistent, reflecting a uniform acceptance and expectation of these technologies among health care professionals and patients. The consensus on telemedicine’s future role over the next 5 years indicates a unified vision for digital health paradigms. These consistencies between the 2 groups might be future drivers for improvements in accessibility, convenience, and efficiency in health care delivery.

## Introduction

Digital innovation in health care is accelerating in growth and presence in the media, while the regulatory framework is constantly evolving and adopting. Digitization has found its role in everyday medical life. It is of paramount importance to understand its use and acceptance to improve and continuously develop technology-based health offerings.

First of all, it makes sense to define the term *digital health*: in the search for a unified definition, diverse terminologies and conceptual frameworks are identified in the literature. Fatehi et al [1] conducted a quantitative analysis and term mapping of 95 unique definitions, revealing that digital health is predominantly concerned with the provision of health care rather than the mere use of technology. Their findings highlight a focus on the well-being of individuals at both population and individual levels, emphasizing the use of data and information to enhance patient care. The prevalence of mobile health (mHealth) as a dominant concept within digital health ecosystems was evident, closely interlinked with telehealth and artificial intelligence (AI) applications [1]. In turn, digital health can be subcategorized into a variety of dimensions. eHealth (and its subset mHealth) serves as a foundational category, in which health information technology plays a critical role, focusing on the management and exchange of health information through electronic health records and interoperability systems [2,3]. In the following, these definitions will be assumed for this study.

Developments have been catalyzed by the global COVID-19 pandemic situation [4] and by early legislation in favor of digital health applications (eg, in Germany under the acronym DiGA [*Digitale* *Gesundheits**a**nwendungen*]) [5]. Many promises—such as the availability of telemedicine—have been met, while specific needs regarding data storage are strongly underserved [6]. The use and development of digital health applications differ in addressing the needs of professionals versus those of patients. Acceptance is influenced by factors varying in complexity and depth, often driven by fears or lack of understanding. National legal and technical regulations have yielded a desired increase in the development and usage of digital health care solutions. The underlying study examines the current status and the drivers and barriers of this development, which have been found to be major influencing factors in the adoption of digital technologies in health care [7]. Specifically, it analyzes the gaps in the aforementioned categories between patients and professionals. Additionally, it helps understand the current and future needs of digital health and the requirements for offered applications.

A multinational survey among health care professionals and patients focused on this delta, its underlying reasons, and the consecutively arising demand specifically regarding the following five research questions:

1. How do estimations of the future importance of digital health differ between patients and professionals?
2. How do patients and professionals estimate their future use of digital health solutions?
3. Which areas offer specific innovation potential in digital health from the perspective of patients and professionals?
4. Which key obstacles to using digital health solutions do patients and professionals identify?
5. How does the use of web-based access differ toward health data, electronic appointment booking, and telemedicine?

Every nonmedical professional from the general population is considered a patient for research purposes.

## Methods

### Web-Based Survey

An anonymous web-based survey, adopted from some of the authors’ [FS, VF, GME, DP] earlier research in 2018 [8], was conducted between April and September 2023 via the scientific survey platform SoSci Survey [9] hosted on German servers complying with European data protection laws. The survey was initiated with an introductory question distinguishing between patients and physicians, deciding on the further direction of the questionnaire. Depending on the chosen branch, 19 questions for patients and 25 for physicians followed, 3 of which were open-text questions. The questionnaire was provided in German and English and was available for 6 months: 6 April through 30 September 2023. Only fully completed questionnaires were considered for final analysis to guarantee consistent data quality.

The link to the web-based survey was distributed via publications in scientific journals such as *Die Unfallchirurgie*, the networks of the German Society for Orthopaedic and Trauma Surgery (*Deutsche Gesellschaft für Orthopädie und Unfallchirurgie*) and the German Society for Surgery (*Deutsche Gesellschaft für Chirurgie*), and web-based communication platforms such as LinkedIn and university web pages to reach a broad range of participants in both the professional field and among patients.

Participants were asked a set of specific questions regarding their personal and professional use, their acceptance and future expectations toward digital health care, followed by sociodemographic questions, and questions regarding their field of expertise, age, and country of origin. In addition, open-text questions were offered to understand and evaluate individual needs and gain additional insights. The English version of the survey is in Multimedia Appendix 1.

In this study, a quantitative analysis was conducted using the Python programming language, leveraging the capabilities of the Pandas library for data processing and the Matplotlib library for visualization. This approach allowed for a practical assessment of relative frequencies and distribution patterns in the responses of health care professionals and patients. For binary categorical data, such as responses regarding the “use of electronic appointment system,” chi-square tests were used to assess the association between the respondents’ profession and answers. In cases of ordinal data, including perceptions of the “future importance of digital health” and estimations of telemedicine use in the past and future, the Mann-Whitney *U* test was used. This nonparametric test compares the central tendency of 2 independent samples and is optimally suited for data not conforming to a normal distribution. Responses were numerically coded to facilitate this analysis. The primary objective of this investigation was to compare and contrast attitudes toward and usage of digital health tools, including aspects of telemedicine and web-based health care services. The qualitative data were assessed by a panel of experts (including a physician, a data scientist, and a management expert) and evaluated for underlying trends and overlaps between the questionnaire participants.

### Ethical Considerations

This study was approved by the ethics committee of Klinikum rechts der Isar, Technical University of Munich, before distribution. The approval number is 2023‐146-S-NP. By answering the mandatory questions of the survey and submitting them, the participants provided their consent to the anonymous study. In case they refrained from submitting the answers or refrained from answering specific mandatory questions, their consent was denied. In these cases, data were neither submitted nor recorded in any form. The participants of the survey received no compensation in any form. The privacy and confidentiality of all research subjects' data and identity were maintained at all times, because all data were collected anonymously and no identifying information was submitted at any time.

## Results

### Analysis of the Replies to the Web-Based Survey

During the 178 days of availability, the questionnaire yielded 2058 clicks, of which 1389 visitors answered the questionnaire and became participants in the study (response rate of 67.5% among visitors). A total of 1002 participants (72.1% of the respondents; 947 of whom were German-speaking and 55 of whom were English-speaking) completed the entire questionnaire, and the remaining 387 (27.9%) participants stopped answering the questionnaire before reaching the last question. The resultant dataset comprised 731 patient-derived responses juxtaposed with 271 physician-sourced answers, offering a representative overview of both primary health care stakeholders.

The study revealed a high level of conformity in perspectives between physicians and patients regarding the adoption and future use of digital health tools and telemedicine. Both groups showed parallel levels of adoption for electronic appointment systems and similar historical use patterns of telemedicine offerings. It yielded a comparable evaluation of the future importance of digital health, with no significant differences observed in their responses. Notably, the analysis indicated a consensus in expectations toward future use over the next 5 years, highlighting a synergistic alignment in the outlook toward digital health paradigms. The general attitude toward digital health and telemedicine yielded no significant differences either, underscoring a uniformity in the acceptance and anticipated future use of these technologies among both groups. Future expectations were aligned, with physicians and patients recognizing the importance and potential of digital health solutions in improving health care delivery. This consensus points toward a collaborative readiness for integrating digital health tools in future health care services, emphasizing the transformative potential of digital technologies in the medical field.

When asked about their “web-based access status to the hospital information system,” 66.1% (179/271) of physicians replied positively (“yes”), while 33.9% (92/271) did not have access (“no”). This result refers exclusively to the answers of the doctors and excludes patient data.

As demonstrated in Table 1, the “use of electronic appointment system” elicited a nonsignificant difference in usage patterns between physicians and patients (chi-square test, *P*=.16). This lack of statistical significance indicates parallel levels of adoption between the 2 groups.

**Table 1.** Proportion of health care professionals and patients using the “electronic appointment systems.”

	Yes, n/N (%)	No, n/N (%)	*P* value^a^
Group			.16
Physicians	159/271 (58.73)	114/271 (41.99)	
Patients	454/731 (62.11)	285/731 (38.97)	

a Chi-square test.

The assessment of the perceived “future importance of digital health” is visualized in Table 2. Responses from both cohorts indicated no statistically significant divergence (Mann-Whitney *U* test, *P*=.13), indicating a uniform recognition of the importance of digital health across the medical community and patient population.

**Table 2.** Comparison of the perceived “future importance of digital health” between health care professionals and patients.

	Physicians, n/N (%)	Patients, n/N (%)	*P* value^a^
Response			.13
Very important	181/271 (66.92)	562/731 (76.90)	
Important	65/271 (23.92)	129/731 (17.67)	
Not that important	26/271 (9.43)	47/731 (6.47)	
Not important	5/271 (1.74)	4/731 (0.60)	

a Mann-Whitney *U* test.

As delineated in Table 3, historical use patterns of telemedicine similarly showed no statistical difference between physicians and patients (Mann-Whitney *U* test, *P*=.86), indicating a shared experience regarding the integration of telemedicine into health care delivery over the past 5 years.

**Table 3.** Comparison of responses to “use of telemedicine in the last 5 years” between health care professionals and patients.

	Physicians, n/N (%)	Patients, n/N (%)	*P* value^a^
Response			.86
Increased	214/271 (78.97)	578/731 (79.11)	
Remained the same	53/271 (19.61)	140/731 (19.22)	
Decreased	9/271 (3.14)	22/731 (2.98)	

a Mann-Whitney *U* test.

Prospective inclinations toward “future use of telemedicine in the next 5 years” are graphically represented in Table 4. The analysis revealed no statistically significant difference in expectations between the 2 groups (Mann-Whitney *U* test, *P*=.10), highlighting a consensus in the projected trajectory of telemedicine’s role in future health care services.

**Table 4.** Expectations for the “future use of telemedicine in the next 5 years” among health care professionals and patients.

	Physicians, n/N (%)	Patients, n/N (%)	*P* value^a^
Response			.10
Will increase	229/271 (84.67)	647/731 (88.56)	
Will remain the same	36/271 (13.30)	76/731 (10.46)	
Will decrease	8/271 (2.90)	19/731 (2.59)	

a Mann-Whitney *U* test.

Collectively, the data shows a conformity in perspectives between physicians and patients regarding the adoption and future use of digital health tools and telemedicine. The absence of statistically significant differences underscores a potentially synergistic alignment in the outlook toward evolving digital health paradigms—in other words, a contextual significance with regard to similarities between the 2 groups. Table 5 provides a comparative statistical analysis of responses on digital health adoption between health care professionals and patients using chi-square and Mann-Whitney *U* tests. We used a significance threshold of *P*<.05 to determine whether the differences between the groups were statistically significant. As shown in the table, none of the *P* values for the comparisons (eg, *P*=.16 and *P*=.13) were below this threshold, indicating that there were no statistically significant differences in attitudes between health care professionals and patients. This suggests that both groups share similar perspectives on the various aspects of digital health adoption presented.

**Table 5.** Comparative statistical analysis between the study groups.

Question	Statistical test	*P* value	Significant difference
Use of electronic appointment system	Chi-square	.16	No
Future importance of digital health	Mann-Whitney *U*	.13	No
Use of telemedicine in the last 5 years	Mann-Whitney *U*	.86	No
Future use of telemedicine in the next 5 years	Mann-Whitney *U*	.10	No

### Open-Text Questions

This section summarizes answers to the open-text questions, highlighting the most common answers and key aspects focusing on regulated digital medical products.

#### In Your Opinion, What Can Digitalization Achieve for Patients?

There is a strong emphasis on how digital health solutions can improve patient care and treatment efficiency, with many noting the potential for streamlined treatment processes, quicker and more accurate diagnoses, and a reduction in unnecessary medical tests. Centralized and accessible data management assumes another critical area. Respondents point out the benefits of easily retrievable patient information in an organized format, leading to more informed clinical decisions. This is closely linked with promoting preventive health care and healthy lifestyles, emphasizing the role of digitalization in disease prevention. Reducing the administrative burden on health care professionals is also a significant theme. Digital tools can allow professionals to focus more on direct patient care by minimizing time spent on nonmedical tasks and bureaucracy. Enhancing communication and decision-making between patients and health care providers is viewed as crucial by study participants. Digital solutions are seen as facilitators of patient empowerment and shared decision-making, highlighting the importance of a patient-centered approach in digital health.

Regarding resource management, digitalization is seen as a potential solution to the shortage of health care professionals, supporting more personalized medicine and aiding in health care research through the provision of comprehensive data for analysis. Accessibility and inclusivity pose key themes, with digital health solutions considered vital for making health care more accessible, especially in remote or underserved areas. The responses collectively underscore the transformative potential of digital health solutions in enhancing health care delivery and patient experience.

#### Which Development Potential and Opportunities Do You See in the Digitalization of Health Care?

A recurring theme is the potential for digitalization to reduce bureaucracy and thus significantly improve the health care systems’ efficiency. This aspect is particularly pertinent in the reduction of regulatory complexity associated with digital medical products. Enhanced data management and accessibility emerge as crucial factors, with many responses highlighting the integration of AI in diagnostics and decision-making, the use of big data for predictive health analytics, and the centralization of patient data. These points resonate with the emphasis on using real-world data and evidence for regulatory surveillance and the development of digital health solutions. Improved communication and coordination through digital tools were seen as crucial for better interdisciplinary and interinstitutional collaboration, reflecting the challenges of diverse regulatory landscapes in health care. The attitude toward innovation in medical devices and diagnostics, especially the role of AI and machine learning, the development of new diagnostic tools, and the application of digital twins and simulation techniques, aligns well with the need for reduced regulation of AI products in health care. Patient-centric approaches are emphasized in the responses, highlighting the need for more personalized medicine and improved patient engagement through digital solutions. This underlines the necessary development of useful, patient-centered, regulated medical products. Regulatory strategy considerations, particularly secure data handling and privacy concerns, are implicit in the responses, reflecting the importance of clear regulations in the development and implementation of digital medical products. Lastly, digitalization is viewed as a solution to health care professional shortages and a means to improve health care access, particularly in remote areas.

#### Where Do You See the Largest Risks and Disadvantages of the Digitalization of Health Care?

The responses reveal a multifaceted set of concerns, deeply interconnected with the challenges inherent in regulated digital medical products. A primary worry is data security and privacy, with numerous responses highlighting apprehensions about the misuse of personal health information. This emphasizes the need for stringent regulatory and ethical frameworks in the development and deployment of digital medical tools, as perceived by the study participants. Another significant concern is the depersonalization of care. Many participants fear that the rise of digital therapeutics and diagnostics might erode the personal interaction between health care providers and patients, a perceived cornerstone of adequate health care. Balancing technological efficiency with human empathy is, therefore, a key challenge in the realm of digital health. Technological dependency and system vulnerabilities emerge as major concerns. Respondents worry about an over-reliance on technology, potential system failures, and cybersecurity threats, emphasizing the importance of safety and reliability in digital medical products, especially in critical care. Resistance to change is another notable theme, particularly among health care professionals and older patients. This resistance can significantly impact the adoption and effectiveness of digital health solutions. Finally, navigating the complex regulatory landscapes and ethical dilemmas, especially with integrating AI and machine learning in health care, presents its challenges. Robust regulatory strategies and ethical guidelines are seen as paramount in ensuring the responsible and effective use of digital health innovations.

### Participant Demography

Considering the age distribution of the participants, the following results were obtained from the questionnaire, as shown in Table 6.

**Table 6.** Age distribution between the 2 study groups. Values are shown as absolute numbers and relative frequencies (percentages in squares) of the whole respective population.

	18‐25 years	26‐35 years	36‐45 years	46‐55 years	56‐65 years	>65 years
Physicians (n=271), n (%)	9 (3.32)	53 (19.56)	64 (23.62)	71 (26.20)	58 (21.40)	16 (5.90)
Patients (n=731), n (%)	35 (4.79)	166 (22.71)	209 (28.59)	155 (21.20)	111 (15.18)	55 (7.52)

## Discussion

### Principal Findings

This survey’s results indicate alignment between professionals and patients regarding the adoption of technological innovations in health care and emphasize the necessity of improving digital health literacy. The continuous growth of digital health technologies is evident [10], with increasing use among professionals [11], the general population, and research [12], including real-world evidence generation [5]. The COVID-19 pandemic has accelerated digital adoption [13], transforming outdated processes and overcoming patient access barriers at the “digital front door.” A balance between “techno-optimism” and “techno-skepticism” has emerged [14].

The study’s open-ended responses reflect concerns about data security, privacy, and ethical issues, with both physicians and patients identifying these as central challenges. The depersonalization of care and ethical dilemmas surrounding digital health innovations remain critical concerns. Systematic reviews confirm that digitalization introduces cybersecurity vulnerabilities, such as targeted cyberattacks and risks to patient privacy. Physicians and patients alike perceive these as barriers to effective implementation [15]. Additional studies highlight the ethical dimensions of digital health, including impacts on the patient-physician relationship, informed consent, and potential workforce shifts. Ongoing research is required to ensure that technological advancements do not compromise patient rights or exacerbate health care inequalities [16].

While many individuals rely on the internet as a primary source of health information and for anonymous consultations, skepticism persists regarding web-based data storage and digital data exchange between health care professionals and patients. Research has shown that, while internet users frequently seek health information on the web, they express doubts about the reliability of such information and the security of electronic health data exchange. Nonetheless, positive developments are also noted: physicians report improvements in access to digital health records compared to previous studies [17].

Despite advancements in digital health applications, integrating patient-generated data presents challenges, particularly due to technological limitations such as poor interoperability and data overload. Experts have emphasized the need for regulatory frameworks, ethical guidelines, and improved data governance to facilitate better integration [18]. Additionally, while protective regulations are necessary to ensure data security, the COVID-19 pandemic demonstrated how excessive regulatory barriers contributed to delays in data availability for policymakers. This underscores the need for streamlined data approval processes while maintaining public trust [19].

Germany has introduced the “Law for the Acceleration of Digitization in Healthcare” (DigiG), currently under legislative review. This initiative aims to implement electronic patient case files, digital medication overviews, electronic medical prescriptions, increased use of digital health applications (DiGAs), assisted telemedicine, and a digital medical council comprising experts in data security, information freedom, and information technology [20]. The governing legal frameworks, particularly the European Union’s Medical Device Regulation, impose comprehensive safety, performance, and postmarket surveillance requirements for digital health applications, ensuring high reliability. However, these rigorous conformity assessments may slow innovation. In contrast, the US Food and Drug Administration framework offers a more expedited approval process for digital health technologies, including AI-driven tools, through mechanisms such as the Software as a Medical Device (SaMD) categorization [21]. This regulatory flexibility has facilitated the rapid adoption of AI in US health care [22].

Research has identified various barriers to digital health adoption, including technical difficulties, resistance to change, costs, reimbursement policies, patient age, and education levels. These findings remain relevant, with technological proficiency, perceived usefulness, and accessibility influencing acceptance worldwide. Additionally, interdisciplinary collaboration, cultural factors, and leadership have been recognized as key enablers of digital health implementation [23,24]. Digital booking systems offer potential benefits, but patient and physician attitudes toward these systems remain understudied [25,26]. Our study demonstrates growing interest in digital health solutions and highlights the necessity for a transparent and innovation-friendly regulatory framework.

Age significantly impacts digital health adoption, as older adults face barriers such as limited digital literacy and reluctance toward technological change [27,28]. These challenges must be addressed through targeted interventions, including comprehensive training programs and the development of user-friendly, age-adapted digital health solutions. Integrating digital health education into medical school curricula is critical to preparing future physicians for evolving technological demands, reducing hesitation, and fostering a legal environment conducive to innovation.

Telemedicine adoption requires specific communication skills to maintain strong physician-patient relationships. A systematic review performed by Ritunga et al [29] highlights that effective digital consultations depend on training that addresses the unique challenges of virtual interactions, such as nonverbal communication and empathy. Additionally, as society shifts toward an information-driven model, medical education must balance humanistic aspects, such as the physician-patient relationship, with digital health integration. Experts warn that without proper integration, medical care quality may decline [30]. These concerns align with this study’s findings, reinforcing the importance of digital health training for both professionals and patients.

Legislative changes, particularly at the European Union level, are essential to facilitate health care’s transition from analog systems to modern digital solutions while ensuring privacy protections [31]. “Careful legal consideration has to be applied when considering privacy regulations (…), making every case an individual one,” keeping the patient at the center not only legally but also ethically, reinforcing the importance of individualized health care approaches.

The increasing alignment between physicians and patients regarding digital health adoption has significant implications for future health care delivery. With both groups recognizing the value of digital health tools and telemedicine, integration into routine practice may become more seamless. This shared understanding could reduce resistance, encourage collaboration in designing patient-centered digital solutions, and enhance accessibility, efficiency, and convenience in health care services.

Further research will enable a deeper understanding of drivers and obstacles in digital health advancements. The underlying research deserves a follow-up including a more international study population in the next few years to understand and re-evaluate the described trend patterns on a timeline, particularly in view of the more and more rapid changes in digital health.

### Limitations

Due to the nature of its origin and the involved researchers, our survey has reached mainly answers from German-speaking individuals (94.5% (947/1002) of the survey’s participants). The survey distribution via digital means causes a bias toward respondents who are open to digital solutions. Persons unwilling or unable to complete a web-based questionnaire were not considered for technical reasons, causing a systematic bias that might potentially be a limiting factor regarding the generalizability of the presented results. Another limiting factor might be the above-mentioned age demographics of the study: as the majority of study participants belonged to the age groups between 26 and 65 years, the study results might lean toward a comparably high digital literacy among the respondents. Additionally, the German or German-speaking study population presents a limitation with regard to the conclusions drawn for a comparison between physicians and patients worldwide. Overall, 271 professionals versus 731 patients is a logical outcome, as all people are patients in general, whereas a smaller percentage counts as medical professionals.

Future research could benefit from exploring subgroup differences, such as attitudes by age, gender, system access, or professional experience, as well as including other health care professionals like nurses, to provide a more nuanced understanding of digital health acceptance. Practical challenges related to accessing individual nurses in this study will be addressed, and a deeper analysis of qualitative responses based on demographic factors could provide additional insights into specific barriers and facilitators.

### Outlook

As also suggested by the replies of the study groups, regulatory aspects must be modified to keep pace with current innovations, allowing for meaningful evaluation, regulation, and dysregulation of new technologies. This aspect is in line with the results of Torous et al [32], who highlighted this urgent need for a steady modification of regulations pertinent to the field of digitalization in health care.

Web-based data availability can lead to misinformation, which needs to be vigorously and continuously counteracted: “To harness the full potential of digital media to support health and well-being as well as to mitigate or counteract the effects of mis- and disinformation, three fundamental skills should be continuously developed: digital literacy, health literacy, and digital health literacy” [33].

In general, perceptions, fears, and resentments must be systematically eliminated to catalyze digital health acceptance and allow for further integration and innovative solutions to benefit patients and professionals. Bureaucratic burdens and technical obstacles must be overcome and transformed from technical barriers into enablers to accelerate acceptance and usage.

Recent developments such as the Data for Health Conference 2023 (DFH23) serve as catalysts for optimistic growth: “With the momentum #DFH23 created, the European Health Data Space (EHDS) as a solid and safe foundation for consented collaborative health data use and the G7 Hiroshima AI process in place, we call on citizens and their governments to fully support the digital transformation of medicine, research and innovation including AI” [34].

Advancements in digital health literacy and enhanced technical reliability will drive openness and catalyze future developments in the patient’s interest and, thus, in the interest of sustainable innovation-driven health care systems.

## Supplementary material

10.2196/60779 Multimedia Appendix 1 Questionnaire.

## Abbreviations

AI: artificial intelligence
DFH23: Data for Health Conference 2023
DiGA: Digitale Gesundheitsanwendungen
mHealth: mobile health
SaMD: Software as a Medical Device
