# Supplementary material for: Use and Acceptance of Innovative Digital Health Solutions Among Patients and Professionals: Survey Study
Source: JMIR Hum Factors. 2025 May 8;12:e60779. doi: 10.2196/60779 (PMC12080968; doi:10.2196/60779)
Supplement: Multimedia Appendix 1 [file humanfactors-v12-e60779-s001.docx]

**Multimedia Appendix 1: Questionnaire**

Questions are directed exclusively at professionals in the healthcare sector; questions are directed exclusively at patients.

1. Are you a medical doctor?

(Yes/No): A=yes; B=no

1. How often do you use digital medical software offerings (e.g., medical apps / Digas (digital health applications))?

(Likert scale): 1=never; 2=very rarely; 3=often; 4=regularly

1. How often do you use electronic hardware for medical purposes (e.g., wearables)?

(Likert scale): 1=never; 2=very rarely; 3=often; 4=regularly

1. Which of the following mobile devices do you use in your daily clinical routine, for example, during ward rounds / in patient contact?

(Multiple selections possible): A=private device; B=device provided by the clinic; C=both; D=I do not use mobile devices for medicine

1. Do you have online access to the HIS (Hospital Information System) of your facility?

(Yes/No): A=yes; B=no

1. Do you use an electronic appointment scheduling system for organizing patient appointments?

(Yes/No): A=yes; B=no

1. Do patients or referring physicians - including those within the hospital - have the option to directly access this electronic calendar online?

(Yes/No): A=yes; B=no

1. Do you use an online platform or cloud solution to store your personal health data?

(Yes/No): A=yes; B=no

1. Do your patients use an online platform or a cloud solution to store their personal health data?

(Likert scale): 1=none; 2=only a few; 3=many; 4=most of them

1. As a medical doctor, do you download or use clinical data of your patients from other medical facilities outside your organization?

(Likert scale): 1=never; 2=very rarely; 3=often; 4=regularly

1. How do you assess the current significance of digital healthcare?

(Likert scale): 1=not important; 2=not that important; 3=important; 4=very important

1. How do you assess the future significance of digital healthcare?

(Likert scale): 1=not important; 2=not that important; 3=important; 4=very important

1. How do you assess the use of telemedicine in the last five years?

(Single choice): 1=Increased; 2=decreased; 3=remained the same

1. How do you think the use of telemedicine will evolve in the next five years?

(Single choice): 1=Will increase; 2=will decrease; 3=will remain the same

1. What is your personal attitude towards digital healthcare?

(Single choice): 1=Critical; 2=neutral; 3=open; 4=very willing to use

1. Where do you see the greatest potential for innovation in the field of digital

healthcare?

(Multiple selections possible): A=Papers and documentation; B=storage of patient data; C=analysis of patient data; D=treatment decisions; E=robotics

1. Where do you see the most significant obstacles / hurdles for innovations in the field of digital health?

(Multiple selections possible): A=Economic; B=societal; C=psychological; D=technical; E=medical; F=legal/regulatory; G=ethical; H=other (free text)

**Open text questions:**

1. In your opinion, what can digitalization achieve for patients?
2. Which development potentials and opportunities do you see in the digitalization of healthcare?
3. Where do you see the largest risks and disadvantages of the digitalization of healthcare?
4. How and where do you believe personal health data should be stored?

(Multiple selections possible): A=electronic health card or electronic health record; B=medical facility (e.g., family doctor or hospital); C=insurance; D=private cloud storage; E=commercial provider

1. Personal Information: Gender

(Single choice): A=female; B=male; C=non-binary

1. Personal information: Educational level

(Single choice): A=Resident; B=specialist; C=consultant; D=chief consultant

Personal information: Educational level

(Multiple selections possible): A=Secondary school (German Hauptschule); B=secondary school with a more advanced curriculum (German Realschule); C=general university entrance qualification (German Gymnasium; equivalent to high school diploma); D=vocational school; E=university studies; F=doctorate (PhD)

1. Personal information: Medical field

(Multiple selections possible): A=Anaesthesiology / emergency medicine; B=cardiology; C=dermatology; D=ear, nose, and throat; E=general medicine; F=general surgery; G=gynaecology; H=cardiac and thoracic surgery; I=internal medicine; J=microbiology / hygiene; K=nephrology; L=urology; M=oncology; N=orthopaedics / trauma surgery; O=pathology / histology; Q=psychiatry and psychotherapy; R=radiology; S=vascular surgery; T=neurology; U=neuro surgery; V=; no patient-related activity X=other (free text input)

1. Personal information: Type of occupation

(Single choice): A=Clinic; B=private practice; C=researcher

Personal information: Type of occupation

(Single choice): A=Unemployed/job seeker; B=employee/civil servant; C=self-employed; D=retired/pensioner

1. Personal information: Age

(Single choice): A=18-25; B=26-35; C=36-45; D=46-60; E=older than 60
